# Is Dexamethasone Helpful in Reducing Perihematoma Edema and for the Outcome of Intracerebral Hemorrhage?

**DOI:** 10.3390/jcm15010352

**Published:** 2026-01-02

**Authors:** Jayantee Kalita, Sandeep Kumar Gupta, Dhiraj Kumar, Firoz M. Nizami, Prakash C. Pandey, Roopali Mahajan, Vivek Singh

**Affiliations:** 1 Department of Neurology, Sanjay Gandhi Post Graduate Institute of Medical Sciences, Raebareli Road, Lucknow 226014, Uttar Pradesh, India; sandeepngci@gmail.com (S.K.G.); dhiraj05rims@gmail.com (D.K.); mdfiroznizami@gmail.com (F.M.N.); drprakashpandey@gmail.com (P.C.P.); mahajanroopali007@gmail.com (R.M.); 2 Department of Radiodiagnosis, Sanjay Gandhi Post Graduate Institute of Medical Sciences, Raebareli Road, Lucknow 226014, Uttar Pradesh, India; singhvivek79@rediffmail.com

**Keywords:** dexamethasone, stroke size, perihematoma edema, hematoma edema complex, mortality

## Abstract

**Background:** In primary supratentorial intracerebral hemorrhage (PSICH), dexamethasone (Dexa) may be effective in reducing perihematoma edema (PHE). We compare the changes in the PHE, hematoma edema complex (HEC), and midline shift (MLS) in patients with PSICH in the Dexa and Non-Dexa groups. **Methods:** The CT-proven PSICHs were included, and their stroke risk factors, Glasgow Coma Scale (GCS) score, and National Institute of Health Stroke Scale (NIHSS) score were noted. Thirty-one patients received intravenous dexamethasone from day 4 to day 7 of stroke in a dose of 24 mg, 12 mg, and 8 mg daily for 3 days each. Thirty-three patients did not receive dexamethasone. The primary outcome was the change in PHE, HEC, and MLS at 15 days compared to the pre-Dexa CT scan, and the secondary outcomes were death and disability at 3 months and side effects. **Results:** The Dexa group had a higher volume of ICH, HEC, and PHE, and MLS compared to the Non-Dexa group, although their age, NIHSS and GCS scores were comparable at admission and just before intervention. The Dexa group had a larger reduction in HEC (*p* = 0.03) and MLS (*p* < 0.01) compared to the Non-Dexa group. The change in PHE volume was also insignificantly higher in the Dexa group (*p* = 0.36). At 3 months, the patients with medium (*p* < 0.001) and large-size hematomas (*p* < 0.001) in the Dexa group had a good outcome, but this benefit was not observed in small hematomas. **Conclusions:** In PSICH, dexamethasone after 3 days reduces the HEC and MLS and may have survival and disability benefits especially in medium and large hematomas. A multicentric–randomized–controlled trial may confirm these findings.

## 1. Introduction

Intracerebral hematoma (ICH) constitutes 20% of all strokes, and about 3 million people in the world die of ICH each year [1]. Only 25% of survivors can walk independently at 6 months [2]. Following ICH, there is a cascade of events resulting in clinical deterioration, higher mortality, and disability. Perihematoma edema (PHE) develops as a result of an increase in extracellular water adjacent to the hematoma and has been attributed to thrombin activation, blood-brain–barrier (BBB) breakdown, the cytotoxic effect of hemoglobin products, and inflammatory immune responses [3,4]. Temporal kinetics of PHE have been reported in earlier studies. In human studies, PHE has been demonstrated within 1–3 days and peaks around 1–2 weeks; thereafter, it declines gradually [5,6,7]. The larger the hematoma, the larger the PHE [5]. The results of the surgical evacuation of deep-seated ICH are not promising [8,9]. The recent MIND trial also did not reveal 30-day mortality and 180-day disability benefits following minimally invasive surgery compared to medical management alone in the patients with spontaneous supratentorial ICH [10]. Therefore, various other treatments have been explored in experimental studies to reduce the PHE, including osmotic agents (mannitol, hypertonic saline), anti-inflammatory drugs (celecoxib, dexamethasone, diethyl fumarate, fingolimod, siponimod, edaravone, glibenclamide, memantine, minocycline, monascin), therapeutic hypothermia, tolvaptan and erythrocyte degradation products (desferioxamine) [11,12,13]. Osmotic therapies, including mannitol, hypertonic saline, and glycerin may reduce PHE temporarily, but these substances are also leaked into the perihematoma region due to BBB permeability, leading to more edema and a midline shift after a transient improvement [14,15,16]. Moreover, osmotic reagents do not address the mechanism of brain edema. In the rat-collagenase model of ICH, intraperitoneal dexamethasone decreased ICAM-1 (intracellular adhesion molecule-1), MMP-9 (matrix metalloproteinase-9) and PHE [17].

A systematic review of dexamethasone, including 490 ICH patients from seven randomized–controlled trials (RCT) revealed no significant benefit or harm. These studies were based on a small number of patients, with introduced dexamethasone from the day of admission, including both supra and infratentorial ICH, and the endpoints of the outcomes were heterogeneous [18]. These studies did not evaluate the effect of dexamethasone on the PHE and hematoma edema complex (HEC). We could not obtain a human study evaluating the effect of dexamethasone on the PHE and HEC in primary supratentorial ICH (PSICH). We hypothesize that a delayed initiation of dexamethasone at the time of the maximum proinflammatory cytokine response may be able to reduce the immune-mediated PHE and HEC; thereby, it may reduce mortality and improve the functional outcome of ICH patients. In this study on PSICH, we report the effect of a short course of delayed dexamethasone therapy on the PHE and HEC compared to those who did not receive dexamethasone. We also compare the short- and long-term outcomes between the two groups.

## 2. Subjects and Methods

This is a propensity matched age, Glasgow Coma Scale (GCS) score, and National Institute of Health Stroke Scale (NIHSS) score case–control study conducted in a tertiary care teaching hospital. The patients were included from a study cohort of a randomized–controlled trial [19]. The Institute Ethics Committee approved the study protocol (Ethics no. 2022-20-IMP-125), and the patient or their caregiver gave consent.

### 2.1. Inclusion Criteria

**Figure 1:**
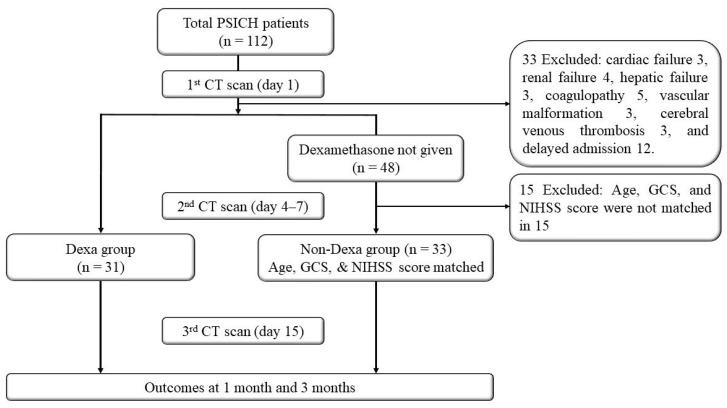
Flowchart.

During the study period, 112 patients with PSICH were admitted within 48 h of ictus. Thirty-one patients received dexamethasone a median of 5 days after stroke onset. From the remaining cohort who did not receive dexamethasone, 33 age-, GCS score-, and NIHSS score-matched patients were included for comparison (Non-Dexa group; Figure 1).

### 2.2. Exclusion Criteria

Intracerebral hematoma secondary to trauma, vascular malformation, bleeding or coagulation disorders, tumor bleed, malignancies, cerebral venous thrombosis, subarachnoid hemorrhage, isolated intraventricular hemorrhage, and central nervous system infections were excluded. Patients on antiplatelet or anticoagulant therapy, children (below 18 years), elderly (above 80 years), and those with pregnancy, lactation, immunosuppressive treatment, or organ transplantation were excluded. Denial of consent by the patient or their caregivers was also an exclusion criterion.

### 2.3. Clinical Evaluation

A detailed history and clinical examination were conducted. The demographic details, stroke risk factors (hypertension, diabetes, hyperlipidemia, sedentary lifestyle, smoking, and tobacco chewing), focal deficits, altered sensorium, seizure, extensor posturing, hyperventilation, blood pressure, and heart rate were noted. Consciousness was assessed using the GCS and stroke severity by the NIHSS.

### 2.4. Investigations

Hemoglobin, blood counts, blood glucose, serum creatinine, albumin, calcium, bilirubin, and transaminases were measured. Systematic inflammatory response syndrome (SIRS) was considered if 2 of the 4 following criteria were fulfilled: (a) fever (>38 °C) or hypothermia (<36 °C), (b) tachycardia (>90/min), (c) tachypnoea 20 breaths/ minute, and leukocytosis (>12,000/mm^3^) or leucopenia (<4000/mm^3^) [20]. The cranial CT scan was performed using a third-generation CT scanner, and slices were obtained at 5 mm thickness (Philips Ingenuity Core 128, Philips Medical System, Best, The Netherlands, 2018). The location and size of the hematoma, intraventricular extension, and midline shift (MLS) were noted. For measuring MLS, a line was drawn joining the most anterior and posterior visible points of the falx. A perpendicular distance from this line to the septum pellucidum in millimeters was considered as MLS [21]. The volume of hematoma, HEC, and PHE was measured by clip and 3D segmentation using Philips IntelliSpace Portal Version 12.1. This is a semi-automated method in which the evaluator outlined the region of interest (hematoma and PHE) in the slice, following which the automated software calculated the volume [22].

All the patients underwent a repeat CT scan on day 7 or earlier if clinically indicated, and after the 7th day of intervention. The above-mentioned parameters were also recorded in the repeat CT scan.

### 2.5. Treatment

The patients were admitted to the intensive care unit if they needed assisted ventilation or in high density unit. All patients had continuous monitoring of blood pressure, pulse, respiratory rate, oxygen saturation, and electrocardiogram using a cardiomonitor. Antihypertensives were prescribed if the admission blood pressure was more than 160/100 mmHg. After 15 days of post-ictus, the target BP was ≤140/90 mmHg. Patients with diabetes received insulin on a sliding scale, until their clinical status improved to some extent. Patients with diabetes and those in the Dexa group had blood glucose measurements every 6 hours, and if the blood glucose was more than 160 mg/dL, subcutaneous insulin was prescribed using a sliding scale. Thereafter, they were switched over to oral antidiabetic drugs (metformin, glimepiride, or vildagliptin) or continued insulin, depending on their glycemic status. Patients having clinical evidence of raised intracranial pressure (pupillary asymmetry, hyperventilation, and extensor posturing) received 100 mL of 20% mannitol intravenously, as and when required. Patients with impending respiratory failure or hypoxia, acidosis, or hypercarbia on arterial blood gas analysis were intubated and mechanically ventilated. However, MV patients were not hyperventilated to reduce the raised intracranial pressure. No patient had a surgical evacuation of the hematoma or extra ventricular drainage of cerebrospinal fluid. All patients received supportive care, including fluid and calories. The patients with infection were treated with appropriate antibiotics based on the culture sensitivity or empirically if their blood and body fluid cultures were negative.

Corticosteroid: Intravenous dexamethasone was prescribed to some patients after 4 to 7 days of ictus, at a dose of 2 mL (8 mg) every 8 h for 3 days, then 1 mL (4 mg) every 8 h for 3 days, and 1 mL (4 mg) every 12 h for 3 days. The other patients received standard treatment.

### 2.6. Follow-Up

The GCS score and NIHSS score before the intervention and 7 days (15 days from the ictus) after the intervention were recorded. The functional outcome was evaluated using the modified Rankin Scale (mRS).

### 2.7. Outcomes

The primary outcome was reduction in the PHE and HEC on day 15 after the stroke (7 days after intervention) compared to preintervention. The secondary outcomes were the change in mRS score at 1 month and 3 months and adverse events. The outcomes were categorized as survived versus death and poor (mRS 3–6) versus good (mRS 0–2) for statistical analysis [7].

### 2.8. Sample Size Calculation

There is no study in the literature evaluating the effect of dexamethasone on PHE. We presumed a 20% reduction in the PHE in the dexamethasone (Dexa) group and 10% in the non-dexamethasone (Non-Dexa) group on the day 15 CT scan from the preintervention CT scan (4–7 days of ictus). A total of 54 participants were required (27 in each group) to detect a statistically significant difference with 95% power at a two-sided alpha level of 0.05. Allowing for an anticipated 10% dropout rate, the final sample size was adjusted to 60 participants. The sample size was calculated using G*Power software (version 3.1.9.7; Universität Düsseldorf, Germany).

Statistical analysis: The heterogeneity of data was checked using the Shapiro–Wilk test. The baseline characteristics between the Dexa and Non-Dexa groups were compared using chi-square for categorical, independent *t*-test for continuous normally distributed data, and Mann–Whitney U test for skewed data. The intergroup and intragroup comparison of GCS score, NIHSS score, and CT scan volumetric parameters (hematoma, PHE, HEC, and MLS) at admission, just before intervention, and 1-week post-intervention were conducted using the chi-square/Fisher’s exact test for categorical data, independent *t*-test for normally distributed continuous data, and the Mann–Whitney U test for skewed continuous variables. The volume of hematoma, PHE and HEC, and MLS on day 7 of Dexa treatment was compared with the Non-Dexa group using either the independent-t test or the Mann–Whitney U test. The outcomes at 1 month and 3 months between the Dexa and Non-Dexa groups were evaluated using the chi-square/Fisher’s exact test. Kaplan–Meier survival curves were plotted for the Dexa and Non-Dexa groups. The effect size of Dexa on the PHE, HEC, and MLS outcomes was also evaluated. The predictors of death were evaluated using univariate analysis followed by Cox regression analysis. Statistical analysis was performed using SPSS 20 version software, and graphs were prepared using GraphPad Prism 7. A two-sided *p*-value of <0.05 was considered significant.

## 3. Results

Sixty-four patients with PSICH were included, their median age was 55 (range 38–80) years, and 39 (60.9%) were males. Fifty-two patients were hypertensive, 16 diabetic, 26 tobacco chewers, and 19 consumed alcohol occasionally. Thirty-one patients received Dexa, and 33 did not (Non-Dexa). Their baseline age, gender, GCS score, NIHSS score, and biochemical parameters were comparable. The Dexa group, however, had higher ICH, HEC, and PHE volumes and MLS compared to the Non-Dexa group. The location of ICH, however, was comparable between the two groups. The details are summarized in Table 1.

### 3.1. Comparison of Clinical and CT Scan Parameters Just Before Intervention

The repeat CT scan, after 4–7 days of ICH, revealed significantly higher volumes of hematoma, HEC, and PHE in the Dexa group compared to the Non-Dexa group. The MLS was also higher in the Dexa group compared to the Non-Dexa group (median 6 vs. 2 mm; *p* < 0.001). The comparison of the clinical and CT scan parameters just before Dexa is presented in Table 2.

### 3.2. Effect of Dexa on Clinical and CT Scan Parameters

**Figure 2:**
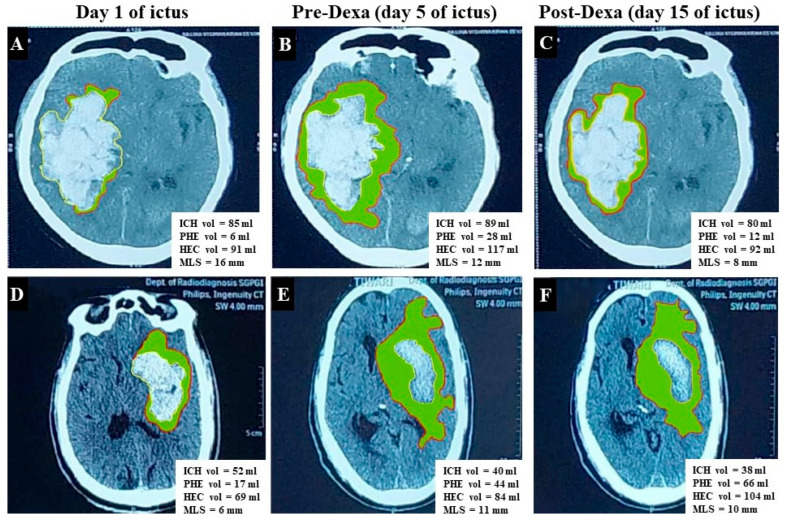
CT scan shows change in volumes of intracerebral hematoma (ICH), hematoma–edema complex (HEC), perihematoma edema (PHE) [Green shaded area represents PHE], and midline shift (MLA). (**A**–**C**) Cranial CT images are of a 75-year-old male with primary supratentorial ICH (PSICH) and show the effect of dexamethasone. There is increase in the volumes of ICH, PHE, HEC, and MLS on the second CT (**B**) compared to baseline (**A**). The HEC, PHE, and MLS decreased on the day-15 CT scan (**C**–**E**), and (**F**) presents the CT scan image of a 72-year-old male with PSICH who did not receive dexamethasone. There is a reduction in the ICH volume, but the HEC and PHE increased on day 15.

Intra-group comparison: In the Dexa group, the volumes of ICH, HEC, and PHE reduced significantly after 7 days of treatment. The MLS also reduced significantly. These were associated with improvements in the GCS score and NIHSS scores. The Non-Dexa group also showed similar improvements, except for the PHE volume (*p* = 0.07). The details are presented in Table 3 and Figure 2.

### 3.3. Primary Outcomes

The Dexa group had a significantly larger change in the volume of HEC [median 7.0 mL (Q1, 5–Q3, 10) vs. median 5.0 mL (Q1, 3.0–Q3, 7.0); *p* = 0.046)] and MLS [median 4.0 mm (Q1, 1.0–Q3, 5.0) vs. 0.0 mm (Q1, 0.0–Q3, 2.0); *p* < 0.01] compared to the Non-Dexa group. The percentage change in PHE volume in the Dexa group was 33.0 (Q1, 0.0–Q3, 50.0) and in the Non-Dexa group 25.0 (Q1, −20.0–Q3, 43.7); *p* = 0.30. The change in the absolute PHE volume was also insignificantly higher in the Dexa group (median 2.0 vs. 1.0; *p* = 0.26). However, the changes in the GCS and NIHSS scores were not significant between the Dexa and Non-Dexa groups. The details are presented in Table 4. The effect size of Dexa on the change in HEC was 0.31, on PHE, 0.24, and on MLS, 0.88.

### 3.4. Secondary Outcomes

**Figure 3:**
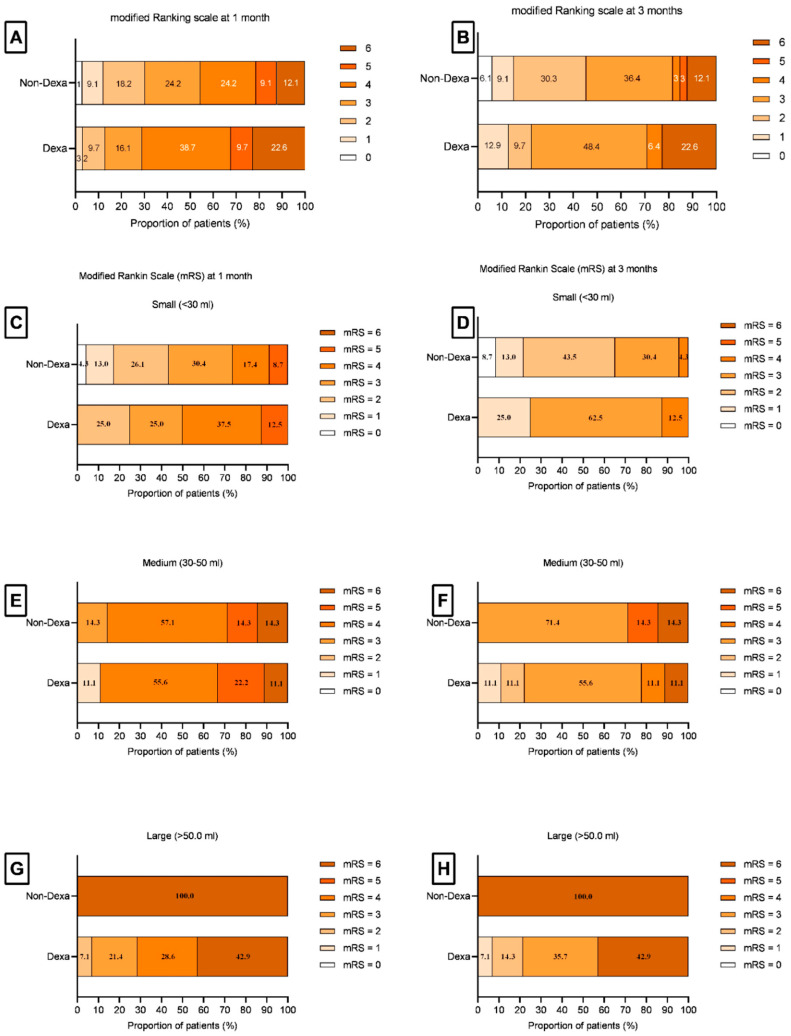
Bar diagram shows the modified Rankin Scale (mRS) scores at 1 month and 3 months. (**A**,**B**) show the percentage of total patients between the Dexa and Non-Dexa groups at 1 month (**A**) and 3 months (**B**). (**C**–**H**) show the percentage of patients having an mRS score in small (**C**,**D**), medium (**E**,**F**), and large (**G**,**H**) hematomas at 1 month and 3 months. The medium and large size hematoma patients in the Dexa group had survival and disability benefits.

Ten (15.6%) patients died at discharge, and one more died after one month. At 3 months, 42 (65.6%) had poor recovery, and 22 (34.4%) had good recovery. The death at 1 month (7, 63.6% vs. 4, 36.4%; *p* = 0.33) and poor outcome (including death) at 3 months (24, 57.1% vs. 18, 42.9%; *p* = 0.07) were not significantly different between the Dexa and Non-Dexa groups (Table 5), although the ICH volume was larger in the Dexa group. At 1 month, the survival benefit of Dexa was observed in the large ICH (>50 mL) compared to the Non-Dexa group [8 (100.0%) vs. 0 (0.0%); *p* < 0.001]. At 3 months, a higher proportion of medium (*p* < 0.001) and large hematomas (*p* < 0.001) in the Dexa group had a good outcome; whereas a higher proportion of small hematomas had a poor outcome (*p* < 0.001) (Figure 3; Supplementary Table S1). The stroke risk factors between the Dexa and Non-Dexa groups were comparable among the patients with medium and large hematomas. The patients with small hematomas in the Dexa group had infections more frequently compared to the Non-Dexa group [3 (21.4%) vs. 0.0 (0.0%); *p* = 0.01]. Amongst the patients with infection, seven out of fourteen patients in the Dexa group and four out of eight patients in the non-Dexa group died (*p* = 1.00).

On univariate analysis, death was associated with the admission GCS score, NIHSS score, serum creatinine, SIRS, ICH, HEC, PHE volumes, and MLS. On Cox regression analysis, the independent predictors of death were the GCS score (adjusted odds ratio 0.63, 95% CI 0.41–0.97, *p* = 0.03) and the NIHSS score (adjusted odds ratio 1.2, 95% CI 1.05–1.38, *p* = 0.009). The Kaplan–Meier survival curve of the Dexa and the Non-Dexa groups is presented in Supplementary Figure S1.

### 3.5. Adverse Events

The Dexa group had insignificantly higher infections and an increase in blood sugar and blood pressure compared to the Non-Dexa group (Table 6). During the post intervention 7 days, the highest systolic blood pressure [median 164.0 (152.0–177.0) vs. 163.0 (150.0–170.0); *p* = 0.90] and diastolic blood pressure [median 120.0 (Q1, 110.0–Q3, 130.0) mm of Hg vs. 120.0 (110.0–130.0) mm of Hg; *p* = 0.30] was comparable between the Dexa and the Non-Dexa groups. The variability in the systolic (*p* = 0.41) and diastolic blood pressure (*p* = 0.66) was also not significant between the two groups. However, the highest blood sugar level [median 198.0 (146.0–259.0) vs. 151.0 (136.0–172.0); *p* = 0.04] was higher in the Dexa group compared to the Non-Dexa group (Table 6). Six patients in the Dexa group needed augmentation of insulin or oral hypoglycemic drugs, with two in the Non-Dexa group (19.4% vs. 6.1%, *p* = 0.14). Two patients in the Dexa group required additional antihypertensive drugs, whereas none did in the Non-Dexa group (6.5% vs. 0.0%, *p* = 0.23).

## 4. Discussion

In the present study, a short course of dexamethasone from days 4 to 7 of stroke resulted in a reduction in the HEC and MLS on day 15 compared to those who did not receive dexamethasone. Patients on dexamethasone also had a higher possibility of survival at discharge and a good outcome at 3 months, especially in the patients with large and medium size hematomas. These benefits were observed despite higher volumes of ICH, HEC, and MLS at baseline in the Dexa group. These results are in concordance with the study by Lu et al. [23]. They evaluated 3214 patients clinically; of whom 529 received Dexa, and 2685 did not. Dexamethasone had a robust effect in reducing short-term mortality in critically ill ICH (non-traumatic) patients, but there was no benefit on the long-term survival. At 1 month, the Dexa group had 8.1% mortality compared to 19.1% in the Non-Dexa group. After propensity matching, the Dexa group had a longer hospital stay (12.7 days vs. 8.8 days, *p* < 0.05) and mortality reductions until one year compared to the Non-Dexa group. They found low-dose Dexa (<12 mg/d) more effective than high-dose (>12 mg/d) in reducing death [23]. However, the day of initiation and duration of dexamethasone and the CT scan findings are not mentioned. We prescribed dexamethasone after a median of 5 (range 4–7) days of stroke. The repeat CT scan was performed based on clinical need; therefore, there was variability in the initiation of Dexa. A larger hematoma likely to have more HEC and PHE in a follow-up CT scan within 7 days compared to a small hematoma and was likely to deteriorate [7,24,25]. The propensity match in our study was based on age, GCS score, and NIHSS score, because these clinical parameters have the strongest role in predicting death and disability. In spite of the larger volume of ICH in the Dexa group, these parameters were not significantly different between the two groups, although the GCS score was lower, and the NIHSS score was higher insignificantly in the Dexa group. The GCS and NIHSS are scales rather than absolute values; moreover, the neurological deficit is highly influenced by its strategic location rather than the volume of stroke [26].

Three meta-analyses have evaluated the effect of corticosteroids in the patients with primary ICH. Feigin et al. evaluated five studies, including 206 patients with primary ICH. Overall, there was no beneficial or adverse effect of corticosteroids in ICH. The authors highlighted the heterogeneity in the study design, study population, type of corticosteroid, and outcome measures [18]. A subsequent meta-analysis by Wintzer et al. also reported a lack of clear benefit of Dexa in reducing death or poor outcomes in patients with spontaneous ICH [27]. Wang et. al. reported a mortality benefit at 3 months in patients with ischemic stroke (31% vs. 26%) and ICH (44% vs. 27%). Corticosteroids, however, did not influence the 6-month outcome either in ischemic stroke or in ICH [28]. There were higher risks of fever, low serum K^+^, and serum protein in the ICH patients treated with dexamethasone [28]. The studies included in these meta-analyses do not include details of the CT scan findings, changes in HEC, PHE, and MLS, or the outcomes in terms of the different sizes of ICH. The exact time of initiation of dexamethasone is not known but is likely to be at the time of stroke. Corticosteroids are effective in vasogenic edema in patients with brain tumors [29,30] and subarachnoid hemorrhage [18,31]. Dexamethasone may be effective when the pro-inflammatory cytokine-mediated PHE increases. Serial CT scans in ICH have revealed a maximum PHE within one to two weeks in the majority of studies; thereafter, the PHE starts declining [6,8,25,30,31,32,33]. The increase in PHE has a linear correlation with the baseline ICH volume [6]. Therefore, a delaying the initiation of dexamethasone for a week may be more beneficial than starting just after the ICH.

Perihematoma edema within a few hours has been attributed to thrombin [34], lysis of erythrocytes, and exudation of plasma. Retraction of the hematoma leads to a reduction in the hydrostatic pressure in the perihematoma region. The exudation of plasma protein leads to a shift in water from the surrounding area, although the BBB in the perihematoma region is intact during this time [35]. Activation of aquaporin-4 allows water leakage, and the failure of Na^+^-K^+^-ATPase results in cytotoxic edema in the hyperacute phase of ICH. During 24–48 h of ictus, there is breakdown of the RBC and activation of thrombin, inflammation, and microglia [36]. At this stage, the BBB breaks down, leading to infiltration of leucocytes, which in turn induces a cascade of inflammatory biomarkers, including nitric oxide, cytokines (TNF-α, IL-6, IL-12), MMP-9, Src kinase, etc. The increase in PHE after 3 days via CT scan is consistent with these underlying pathophysiological mechanisms. This may be the reason why we found survival benefits and better outcomes in our study. Koyuncu et al. showed the effect of dexamethasone in three patients who had delayed deterioration because of increasing PHE. The ages of these patients were 55, 76, and 70 years, and the ICH volumes at ictus were 15 cc, 28 cc, and 15 cc, respectively. They had deterioration on days 12, 16, and 18 and had increased PHE. All these patients had an excellent response to dexamethasone treatment [37].

In our cohort, infection occurred more frequently in the Dexa group compared to the Non-Dexa. Infection is a major problem in intensive care-admitted stroke patients because of multiple intravenous lines, catheter, aspiration pneumonia, or ventilator-associated pneumonia. About 45% patients with stroke develop infections in the intensive care unit [38]. All our patients with infections were admitted to the ICU, and 15 required mechanical ventilation. Infection-related deaths however were comparable between the two groups. The majority of infections are amenable to treatment because of the advancement in the diagnostic methods and antibiotics. Moreover, we used corticosteroids only for 9 days. An earlier meta-analysis did not show an increased risk of infections, except for a few individual studies [28]. In one study, the dexamethasone group had a higher rate of infection (28% vs. 12%) and hyperglycemia (10% vs. 0%) compared to the placebo [39]. The higher death in small hematoma patients in the Dexa group may be due to higher infections compared to the Non-Dexa group. Blood pressure variability has been well documented in patients with stroke and following corticosteroid administration, particularly in the setting of raised intracranial pressure [40]. In our study, both the groups had comparable variability in blood pressure. This may be due to the shorter duration of dexamethasone in a tapering dose and concomitant use of antihypertensive therapy. The Dexa group, however, had higher blood glucose requiring augmentation of insulin in 19.4% patients for a period of 7–14 days.

### Limitations

This was not a randomized–controlled trial, and the day of initiation of Dexa varied between 4 and 7 days (median day 5). The propensity match was conducted on the basis of clinical parameters including age, GCS, and NIHSS and not on the basis of the CT scan findings. The Non-Dexa group also had a larger number of patients with small and lesser large hematoma. At the time of the sample size calculation, we hypothesized that the Dexa group would have 20% reduction in PHE and that of Non-Dexa 10%. The observed group difference of 8% in the PHE might suggest that the true effect of Dexa in the population is smaller than 10%, but it does not change the operational power of the performed test. The biomarkers of inflammation were not measured. The strengths of the study are the prospective data, the measurement of CT scan parameters by a neuroradiologist, and that the clinical findings were verified by a senior neurologist.

## 5. Conclusions

In medium and large ICH, a short course of dexamethasone after day 3 reduces the HEC and MLS, and may have survival and disability benefits, although not in small ICH. A multicentric randomized–controlled trial is needed to confirm these findings.

## Figures and Tables

**Table 1:** Comparison of clinical and investigative findings at admission between Dexa and Non-Dexa groups.

Baseline Parameters		Total (n = 64) Median (Q1–Q3)	Dexa [n = 31 (48.4%)] Median (Q1–Q3)	Non-Dexa [n = 33 (51.6%)] Median (Q1–Q3)	*p*-Value
Clinical and CT scan findings					
Age (years)		55.0 (48.0–65.0)	57.0 (50.0–72.0)	54.0 (46.0–61.0)	0.11
Gender (Male) *		39 (60.9%)	23 (74.2%)	16 (48.5%)	0.04
Risk factors *	HTN	52 (81.2%)	23 (74.2%)	29 (87.9%)	0.21
	DM	16 (25.0%)	7 (22.6%)	9 (27.3%)	0.77
	Smoking	19 (29.7%)	9 (29.0%)	10 (30.3%)	1.0
	Hyperlipidemia	16 (25.0%)	7 (22.6%)	9 (27.3%)	0.77
	Seizure	12 (18.8%)	4 (12.9%)	8 (24.2%)	0.34
	Sedentary lifestyle	27 (42.2%)	15 (48.4%)	12 (36.4%)	0.45
ICH size *	Small (<30 mL)	31 (48.4%)	8 (25.8%)	23 (69.7%)	0.001
	Medium (30–50 mL)	16 (25.0%)	9 (29.0%)	7 (21.2%)	
	Large (>50 mL)	17 (26.6%)	14 (45.2%)	3 (9.1%)	
ICH location *	Lobar	12 (18.8%)	7 (22.6%)	5 (15.2%)	0.20
	Deep	52 (81.2%)	24 (77.4%)	28 (84.8%)	
NIHSS score		18.0 (14.5–25.5)	21.0 (17.0–25.0)	18.0 (13.0–26.0)	0.18
GCS score		11 (9.5–13.5)	11.0 (9.0–13.0)	11.0 (10.0 –14.0)	0.28
LDL (mg/dL)		102.0 (75.5–127.0)	112 (80.0–128.0)	88.0 (69.0–125.0)	0.20
Serum creatinine (mg/dL)		1.11 (0.95–1.40)	1.11 (0.98–1.38)	1.11 (0.92–1.50)	0.89
Serum sodium (mmol/L)		138.0 (133.0–141.0)	139.0 (136.0–144.0)	137.0 (132.0–141.0)	0.11
SIRS		2.0 (2.0–3.0)	3.0 (2.0–3.0)	2.0 (2.0–3.0)	0.15
ICH volume (mL)		30.0 (22.0–51.5)	46.0 (29.0–66.0)	23.0 (20.0–30.0)	<0.001
MLS (mm)		5.5 (3.0–10.0)	7.0 (5.0–13.0)	4.0 (3.0–7.0)	0.001
Absolute PHE (mL)		3.8 (2.0–6.5)	6.0 (3.0–8.0)	3.0 (2.0–4.0)	0.001
Mannitol *		42 (65.6%)	23 (74.2%)	19 (57.6%)	0.19
Metoprolol *		34 (53.1%)	16 (51.6%)	18 (54.5%)	1.00
Amlodipine *		30 (46.9%)	15 (48.4%)	15 (45.5%)	1.00
Mechanical ventilation *		15 (23.4%)	10 (32.3%)	5 (15.2%)	0.14
Duration of hospital stay		17.0 (11.5–23.0)	21.0 (10.0–26.0)	15.0 (12.0–22.0)	0.17

DM = Diabetes mellites; GCS = Glasgow Coma Scale; HTN = hypertension; ICH = intracerebral hemorrhage; LDL = low-density lipoprotein; MLS = midline shift; NIHSS = National Institutes of Health Stroke Scale; PHE = perihematoma edema; SIRS = systemic inflammatory response syndrome. * = n (%).

**Table 2:** Comparison of clinical and CT scan parameters of the Dexa and Non-Dexa group before the initiation of dexamethasone (4–7 days) after stroke onset.

Parameters (4–7 Days)	Dexa [n = 31 (48.4%)] Median (Q1–Q3)	Non-Dexa [n = 33 (51.6%)] Median (Q1–Q3)	*p*-Value
Clinical findings			
NIHSS score	18.0 (15.0–25.0)	14.0 (12.0–20.0)	0.07
GCS score	12.0 (9.0–14.0)	12.0 (11.0–14.0)	0.14
Serum sodium (mmol/L)	138.0 (131.0–144.0)	134.0 (129.0–137.0)	0.047
Serum creatinine (mg/dL)	0.98 (0.87–1.15)	1.0 (0.78–1.6)	0.77
SIRS	2.0 (1.0–2.0)	1.0 (1.0–2.0)	0.32
CT-scan finding			
ICH volume (mL)	48.0 (32.0–72.0)	22.0 (18.0–35.0)	<0.001
HEC volume (mL)	60.0 (38.0–85.0)	28.0 (22.0–42.0)	<0.001
PHE volume (mL)	8.0 (5.0–12.0)	5.0 (4.0–7.0)	0.003
MLS (mm)	6.0 (5.0–12.0)	2.0 (0.0–5.0)	<0.001

GCS = Glasgow Coma Scale; HEC = hematoma–edema complex; ICH = intracerebral hemorrhage; MLS = midline shift; NIHSS = National Institutes of Health Stroke Scale; PHE = perihematoma edema.

**Table 3:** The change in clinical and CT-scan parameters in the patients receiving dexamethasone and those without.

Parameters	Dexa [n = 31 (48.4%)] Median (Q1–Q3)		*p*-Value	Non-Dexa [n = 33 (51.6%)] Median (Q1–Q3)		*p*-Value
	Days 4–7	Days 15		Days 4–7	Days 15	
Clinical findings						
NIHSS score	18.0 (15.0–25.0)	14.0 (11.0–18.0)	<0.001	14.0 (12.0–20.0)	12.0 (9.0–16.0)	<0.001
GCS score	12.0 (9.0–14.0)	13.0 (12.0–14.0)	0.001	12.0 (11.0–14.0)	13.0 (12.0–15.0)	0.007
Serum Sodium (mmol/L)	138.0 (131.0–144.0)	135.5 (131.0–139.5)	0.64	134.0 (129.0–137.0)	134.0 (132.0–139.0)	0.24
CT findings						
ICH vol (mL)	48.0 (32.0–72.0)	40.0 (22.5–52.0)	<0.001	22.0 (18.0–35.0)	18.0 (15.0–30.0)	<0.001
HEC vol (mL)	60.0 (38.0–85.0)	47.0 (25.5–69.5)	<0.001	28.0 (22.0–42.0)	21.5 (18.0–37.5)	<0.001
PHE vol (mL)	8.0 (5.0–12.0)	4.0 (4.0–10.0)	0.03	5.0 (4.0–7.0)	4.0 (3.0–5.5)	0.07
MLS (mm)	6.0 (5.0–12.0)	4.0 (0.0–8.0)	<0.001	2.0 (0.0–5.0)	0.0 (0.0–2.0)	0.002

GCS = Glasgow Coma Scale; HEC = hematoma–edema complex; ICH = intracerebral hemorrhage; MLS = midline shift; NIHSS = National Institutes of Health Stroke Scale; PHE = perihematoma edema.

**Table 4:** Comparison of the change in clinical and CT-scan parameters between Dexa and Non-Dexa groups on day 15.

Parameters	Dexa [n = 31 (48.4%)] Median (Q1–Q3)	Non-Dexa [n = 33 (51.6%)] Median (Q1–Q3)	*p*-Value
CT findings (days 7–15)			
ICH volume (mL)	4.0 (3.0–7.0)	4.0 (3.0–5.0)	0.29
HEC volume (mL)	7.0 (5.0–10.0)	5.0 (3.0–7.0)	0.046
PHE volume (mL)	2.0 (0.0–4.0)	1.0 (−0.5–3.0)	0.26
MLS volume (mL)	4.0 (1.0–5.0)	0.0 (0.0–2.0)	0.001
Clinical findings (days 7–15)			
NIHSS score	2.0 (1.0–4.0)	2.0 (1.0–4.0)	0.96
GCS score	0.0 (−1.0–0.0)	0.0 (−1.0–0.0)	0.65

GCS = Glasgow Coma Scale; HEC = hematoma–edema complex; ICH = intracerebral hemorrhage; MLS = midline shift; NIHSS = National Institutes of Health Stroke Scale; PHE = perihematoma edema.

**Table 5:** Outcome of intracerebral hemorrhagic patients in Dexa and Non-Dexa groups and comparison of proportion of patients with death and disability at 1 and 3 months between the Dexa and Non-Dexa groups in small, medium, and large size hematomas.

Outcome		Total (n = 64)	Dexa [n = 31 (48.4%)]	Non-Dexa [n = 33 (51.6%)]	*p*-Value
At 1 month					
Survived		53 (82.8%)	24 (45.3%)	29 (54.7%)	0.33
Death		11 (17.2%)	7 (63.6%)	4 (36.4%)	
ICH size *					
Small (<30 mL)	Survived	31 (48.4%)	(8) 25.8%	(23) 74.2%	1.0
	Death	0 (0.0%)	(0) 0.0%	(0) 0.0%	
Medium (30–50 mL)	Survived	14 (21.9%)	8 (57.1%)	6 (42.9%)	0.67
	Death	2 (3.1%)	1 (50.0%)	1 (50.0%)	
Large (>50 mL)	Survived	8 (12.5%)	8 (100.0%)	0 (0.0%)	<0.001
	Death	9 (14.1%)	6 (66.7%)	3 (33.3%)	
At 3 months					
Good		22 (34.4%)	7 (31.8%)	15 (68.2%)	0.07
Poor		42 (65.6)	24 (57.1%)	18 (42.9%)	
ICH size *					
Small (<30 mL)	Good	17 (26.5%)	2 (11.8%)	15 (88.2%)	<0.001
	Poor	14 (21.9%)	6 (42.9%)	8 (57.1%)	
Medium (30–50 mL)	Good	2 (3.1%)	2 (100.0%)	0 (0.0%)	<0.001
	Poor	14 (21.9%)	7 (50.0%)	7 (50.0%)	
Large (>50 mL)	Good	3 (4.7%)	3 (100.0%)	0 (0.0%)	<0.001
	Poor	14 (21.9%)	11 (78.6%)	3 (21.4%)	

* Pearson Chi-square/Fisher’s exact test by using percentage of patients.

**Table 6:** Adverse events.

Parameters	Dexa [n = 31 (48.4%)] Median (Q1–Q3)	Non-Dexa [n = 33 (51.6%)] Median (Q1–Q3)	*p*-Value
Hyperglycemia *	3 (9.6%)	2 (6.1%)	0.67
Hypertension *	7 (22.6%)	3 (9.1%)	0.18
Infection *	14 (45.2%)	8 (24.2%)	0.11
Highest random blood sugar (mg/dL)	198.0 (146.0–259.0)	151.0 (136.0–172.0)	0.04
Highest SBP	164.0 (152.0–177.0)	163.0 (150.0–170.0)	0.90
Highest DBP	120.0 (110.0–130.0)	120.0 (110.0–130.0)	0.30
Variability in SBP	47.0 (36.0–60.0)	42.0 (37.0–59.0)	0.41
Variability in DBP	28.0 (20.0–44.0)	27.0 (20.0–30.0)	0.66

SBP = systolic blood pressure; DBP = diastolic blood pressure. * = n (%).

## Data Availability

The data supporting the results of this investigation are available from the first, second, and third authors upon reasonable request.

## Supplementary Materials

The following supporting information can be downloaded at https://www.mdpi.com/article/10.3390/jcm15010352/s1, Figure S1. Kaplan–Meier survival curve between Dexa and Non-Dexa group (A) including total number of patients, (B) medium size hematoma and (C) large size hematoma. Table S1. Comparison of clinical and CT scan parameters between the patients who survived and those who died at 1 month using univariate analysis.
