# Supplementary material for: Is Dexamethasone Helpful in Reducing Perihematoma Edema and for the Outcome of Intracerebral Hemorrhage?
Source: J Clin Med. 2026 Jan 2;15(1):352. doi: 10.3390/jcm15010352 (PMC12787224; doi:10.3390/jcm15010352)
Supplement: Supplementary file 1 [file jcm-15-00352-s001.zip › jcm-4021101-supplementary.pdf]

Table S1. Comparison of clinical and CT scan parameters between the patients who survived and those who died at 1 month using univariate analysis.

**Parameters**<br>Outcome at 1 months		**Survived** [n = 53 (82.8%)]<br>Median (Q1 – Q3)	**Death** [n = 11 (17.2%)]<br>Median (Q1 – Q3)	**p-value**
Age in years		55.0 (49.0 – 67.0)	54.0 (44.0 – 62.0)	0.36
Risk factors*	Hypertension	42 (79.2%)	10 (90.9%)	0.45
	Diabetes mellites	14 (26.4%)	2 (18.2%)	0.72
	Smoking	17 (32.1%)	2 (18.2%)	0.48
	Hyperlipidaemia	11 (20.8%)	5 (45.5%)	0.12
	Seizure	8 (15.1%)	4 (36.4%)	0.20
	Sedentary lifestyle	24 (45.3%)	3 (27.3%)	0.33
ICH size*	<30 ml	31 (58.5%)	0 (0.0%)	<0.001
	30-50 ml	14 (26.4%)	2 (18.2%)	
	>50 ml	8 (15.1%)	9 (81.8%)	
ICH location*	Lobar	8 (15.1%)	4 (36.4%)	0.20
	Deep	45 (84.9%)	7 (63.6%)	
ICH location*	Putamen	8 (15.1%)	0 (0%)	0.41
	Caudate	1 (1.9%)	0 (0%)	
	Thalamus	4 (7.5%)	1 (9.1%)	
	Capsule	3 (5.7%)	2 (18.2%)	
	Lobar	8 (15.1%)	4 (36.4%)	
	Capsulo-thalamic	6 (11.3%)	1 (9.1%)	
	Thalamus-putamen	2 (3.8%)	0 (0%)	
	Capsulo-ganglionic	21 (39.6%)	3 (27.3%)	
LDL (mg/dL)		96.0 (76.0 – 124.0)	116.0 (67.0 – 161.0)	0.29
NIHSS score		18.0 (13.0 – 21.0)	29.0 (27.0 – 32.0)	<0.001
GCS score		12.0 (10.0 – 14.0)	7.0 (6.0 – 9.0)	<0.001
Serum sodium (mmol/L)		138.0 (135.0 – 141.0)	139.0 (131.0 – 146.0)	0.76
Serum creatinine (mg/dL)		1.08 (0.94 – 1.34)	1.40 (1.11 – 1.90)	0.04
SIRS		2.0 (2.0 – 3.0)	3.0 (3.0 – 3.0)	0.007
Metoprolol*		29 (54.7%)	5 (45.5%)	0.74
Amlodipine*		24 (45.3%)	6 (54.5%)	
Baseline CT findings				
ICH vol (ml)		26.0 (21.0 – 42.0)	64.0 (51.0 – 91.2)	<0.001
HEC vol (ml)		30.0 (24.0 – 47.0)	74.0 (55.0 – 95.0)	<0.001
Absolute PHE (ml)		3.0 (2.0 – 6.0)	4.0 (3.8 – 10.0)	0.05
Midline shift (mm)		5.0 (3.0 – 8.0)	13.0 (8.0 – 14.0)	0.001
Change in CT findings (days 7-15)				
ICH vol (ml)		4 (3.0 – 5.0)	5.0 (3.0 – 7.0)	0.70
HEC vol (ml)		6.0 (4.0 – 8.0)	4.0 (3.0 – 7.0)	0.37
Absolute PHE (ml)		1.0 (0.0 – 4.0)	0.0 (-1.0 – 0.0)	0.15
Midline shift (mm)		1.5 (0.0 – 4.0)	-1.0 (-1.0 – 4.0)	0.18

GCS = Glasgow Coma Scale; ICH = Intracerebral hemorrhage; LDL = Low-density lipoprotein; MLS = Midline shift; NIHSS = National Institutes of Health Stroke Scale; PHE = Perihematoma edema; SIRS = Systemic inflammatory response syndrome. * = n (%)

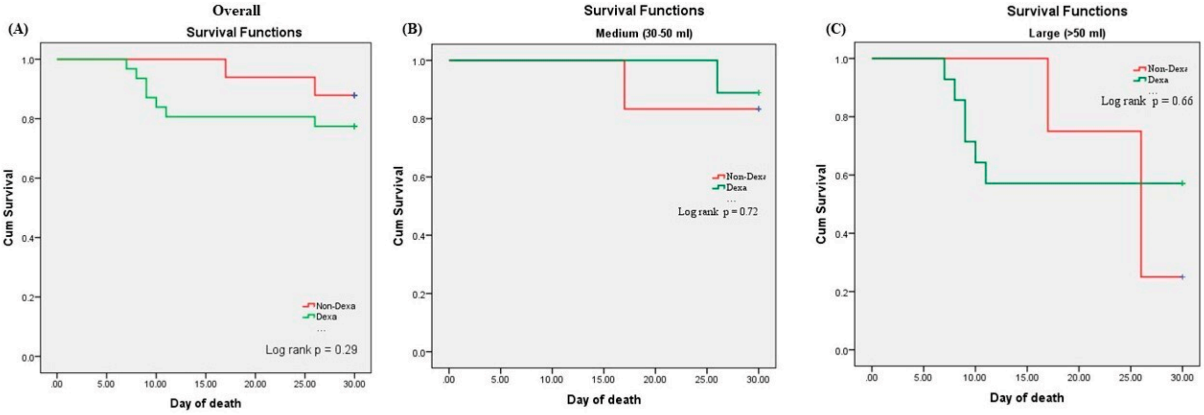

Figure S1. Kaplan–Meier survival curve between Dexa and Non-Dexa group (A) including total number of patients, (B) medium size hematoma and (C) large size hematoma.
